# Supplementary material for: Central Nervous System Progression in Primary Vitreoretinal Lymphoma with Bilateral and Unilateral Involvement: A Systematic Review and Meta-Analysis
Source: Cancers (Basel). 2022 Jun 16;14(12):2967. doi: 10.3390/cancers14122967 (PMC9221335; doi:10.3390/cancers14122967)
Supplement: Supplementary file 1 [file cancers-14-02967-s001.zip › cancers-1688973-supplementary.pdf]

cancers MDPI

Supplementary Material: Central Nervous System Progression in Primary Vitreoretinal Lymphoma with Bilateral and Unilateral Involvement: A Systematic Review and Meta-Analysis

Josephus L. M. van Rooij, Klaudia A. Tokarska, Ninette H. ten Dam-van Loon, Peter H. Wessels, Tatjana Seute, Monique C. Minnema and Tom J. Snijders

Table S1. Search strategy.

Database	Search strategy	Results
**Pubmed**	("VRL"[tiab] OR "PVRL"[tiab] OR (("Lymphoma"[Mesh] OR "Lymphoma*"[tiab]) AND ("Eye"[Mesh] OR "Eye"[tiab] OR "Eyes"[tiab] OR "intraocular"[tiab] OR "Vitreoretinal"[tiab] OR "ocular"[tiab] OR "vitreous retinal"[tiab]))) AND ("Central Nervous System"[Mesh] OR "Central Nervous System Neoplasms"[Mesh:NoExp] OR "Brain Neoplasms"[Mesh] OR "Central Nervous System"[tiab] OR "Central nerve System"[tiab] OR "CNS"[tiab] OR "brain"[tiab] OR "cerebral"[tiab])	**924**
**Embase**	('VRL':ti,ab,kw OR 'PVRL':ti,ab,kw OR (('lymphoma'/exp OR 'Lymphoma*':ti,ab,kw) AND ('eye'/exp OR 'Eye':ti,ab,kw OR 'Eyes':ti,ab,kw OR 'intraocular':ti,ab,kw OR 'Vitreoretinal':ti,ab,kw OR 'ocular':ti,ab,kw OR 'vitreous retinal':ti,ab,kw))) AND ('central nervous system'/exp OR 'central nervous system tumor'/de OR 'brain tumor'/exp OR 'central nervous system cancer'/exp OR 'Central Nervous System':ti,ab,kw OR 'Central nerve System':ti,ab,kw OR 'CNS':ti,ab,kw OR 'brain':ti,ab,kw OR 'cerebral':ti,ab,kw) NOT ('conference abstract'/it OR 'editorial'/it OR 'letter'/it OR 'note'/it) NOT (('animal experiment'/exp OR 'animal model'/exp OR 'nonhuman'/exp) NOT 'human'/exp)	**1195**

Table S2. Customized QUIPS assessment form.

Biases	Issues to consider for judging
Study participation	1. Source of target population<br>2. Method used to identify population<br>3. Recruitment period<br>4. Place of recruitment<br>5. Inclusion and exclusion criteria<br>6. Baseline characteristics<br>7. Timing of diagnosis (before or after CNS progression)
Study attrition	1. Proportion of baseline sample available for analysis<br>2. Reasons and potential impact of subjects lost to follow-up<br>3. Outcome and prognostic factor information on lost to follow-up
Prognostic factor (PF) measurement	1. Proportion of data on PF available for analysis
Outcome measurement	1. Definition of the outcome<br>2. Valid and reliable measurement of outcome<br>3. Method and setting of outcome measurement
Study confounding	1. Important confounders measured<br>2. Definition of the confounding factor<br>3. Appropriate accounting for confounding
Statistical analysis and reporting	1. Presentation of analytical strategy<br>2. Reporting of results
